# Scent-delivery devices as a digital healthcare tool for olfactory training: A pilot focus group study in Parkinson's disease patients

**DOI:** 10.1177/20552076221129061

**Published:** 2022-10-02

**Authors:** Neel Desai, Emanuela Maggioni, Marianna Obrist, Mine Orlu

**Affiliations:** 1 Research Department of Pharmaceutics, UCL School of Pharmacy, University College London, London, UK; 2 Department of Computer Science, 4919 University College London, London, UK

**Keywords:** Digital health, focus group, geriatric, human–computer interaction, non-motor symptoms, olfaction, scent-delivery device, scent training, scent testing, Parkinson's disease, qualitative research

## Abstract

Parkinson's disease (PD) patients display a combination of motor and non-motor symptoms. The most common non-motor symptom is scent (olfactory) impairment, occurring at least four years prior to motor symptom onset. Recent and growing interest in digital healthcare technology used in PD has resulted in more technologies developed for motor rather than non-motor symptoms. Human–computer interaction (HCI), which uses computer technology to explore human activity and work, could be combined with digital healthcare technologies to better understand and support olfaction via scent training – leading to the development of a scent-delivery device (SDD). In this pilot study, three PD patients were invited to an online focus group to explore the association between PD and olfaction, understand HCI and sensory technologies and were demonstrated a new multichannel SDD with an associated mobile app. Participants had a preconceived link, a result of personal experience, between olfactory impairment and PD. Participants felt that healthcare professionals did not take olfactory dysfunction concerns seriously prior to PD diagnosis. Two were not comfortable with sharing scent loss experiences with others. Participants expected the multichannel SDD to be small, portable and easy-to-use, with customisable cartridges to deliver chosen scents and the mobile app to create a sense of community. None of the participants regularly performed scent training but would consider doing so if some scent function could be regained. Standardised digital SDDs for regular healthcare check-ups may facilitate improvement in olfactory senses in PD patients and potential earlier PD diagnosis, allowing earlier therapeutic and symptomatic PD management.

## Introduction

Parkinson's disease (PD) is the most common movement disorder and the second most common neurodegenerative disease,^1^ affecting 1 to 2 persons per 1000,^2^ with that number rising to 1% in the over 60s population^3^ and in the highest age groups, a prevalence of 4%.^4,5^ The critical motor signs and symptoms of PD have been well-documented (e.g. rigidity, slowness of movement and tremor) and are imperative to a PD diagnosis.^1^ Non-motor signs and symptoms are also prominent and have been given considerable clinical research attention in the past decades.^6^

The most common and well-documented non-motor sign is scent (olfactory) impairment, and although generally occurs early in disease progression has been associated with the risk of later clinical PD diagnosis.^7,8^ Olfactory impairment in patients with PD ranges from 75% to 95%^9–12^ compared to 25% in the normal older adult population.^13^ Scent impairment is perhaps the earliest sign of PD neuropathology, with olfactory dysfunction preceding motor signs and symptoms by at least 4 years.^14^ However, some PD patients experience asymptomatic scent loss, which occurs earlier in disease progression,^15^ causing patients to be unaware of the problem.^16^ This results in olfactory impairment being poorly perceived by the PD population. For those PD patients who suffer symptomatic scent dysfunction, there is usually an impact on quality of life. Overall, older adults are more likely to suffer from depressive symptoms when olfactory impairment occurs, as socialising and enjoyment of food and drink become limited.^17^ Olfactory loss is a reliable indicator of early-stage PD^18^; therefore, scent impairment must be considered as a non-motor criterion for PD diagnosis,^19^ with quantification of olfactory function using normalised and standardised tests to report accurate data.^9^

There has been recent and growing interest for technology to improve health-related outcomes. In PD, digital healthcare technologies, a broader term encompassing computing platforms, connectivity, software and sensors for healthcare and related uses,^20^ have mainly been developed to generate accurate, objective and reproducible measurements of motor function,^21,22^ with devices measuring tremor, slowness or involuntary movements, wearing-off, gait patterns, falls and rigidity.^23–25^ These technologies have allowed remote patient observations,^26^ delivery of care without barriers at home and in the community,^27^ reduced the presence of bias in otherwise subjective accounts in PD patient diaries^28^ and are non-disruptive to day-to-day routines.^26^ The field of digital healthcare technologies has also been boosted by the ongoing COVID-19 pandemic.^29^

Olfactory dysfunction has not received the same attention as a target for quantitative measurement using digital healthcare technologies. Human–computer interaction (HCI) technologies have been designed and developed to better understand olfaction, with use limited to academic research, and in some instances, public engagement events and exhibitions. HCI first emerged in the early 1980s and can be defined as the study of how computer technology influences human activities and work.^30,31^ The term covers almost all forms of information technology design and is multidisciplinary, combining computer and cognitive sciences and human factor engineering.^32^ HCI also has an accompanying design component, referred to as interaction design or user-centred design, which focuses on computer technology design to make interactions as easy as possible.^31^

Senses used when interacting with technology are limited, as greater emphasis is placed upon vision, hearing and increasingly touch, with taste and scent largely neglected and under-valued.^33^ The development of HCI technologies for single sensory perception,^34^ either a physical sense (touch, sound or sight) or chemical sense (scent or taste) have been reported to be highly challenging.^35–37^ Despite advancements in sensory system knowledge, the design of multisensory experiences requires careful consideration of the five human senses, how information is processed and the relationship between these interacting senses to fulfil the unmet challenge of understanding people's multisensory HCI experiences.^33^

The perception of scent is complex, yet the olfactory sense is powerful enough to enable humans to react to and act on scent stimuli by discriminating between food or non-food categories^38^ and pleasant and/or unpleasant scents.^39^ Olfactory sensations can influence human body image perception,^40^ feelings about self,^41,42^ and sleep.^43^ Whilst deeper understanding of the human neural circuitry that mediates a sense of aversion or attraction driven by olfaction is still being researched,^44^ the continued attention in scent-scent human–computer interaction technologies has been enhanced by mapping of the olfactory design space using four key features: chemical, emotional, spatial and temporal.^39^ We direct the reader to Cornelio et al.,^45^ for a review on multisensory technology, from which key messages on olfactory HCI technologies are briefly described below.

Some common approaches aimed to present and deliver olfactory cues use analogue methods, including scenting jars of essential oils^46^ and ‘*sniffin'sticks*’, or scented pens^47^; however, these methods fail to provide sufficient control over the delivery of scented stimuli.^45^ The design of more advanced approaches using computer-controlled olfactometers have been trialled but have been found to be bulky, noisy and static.^48,49^

To overcome these challenges, novel scent-delivery devices (SDDs) have been developed by HCI researchers. These SDDs aim to create and/or replicate olfactory experiences by allowing people to perceive a series of singular or mixture of scents and to describe the experience.^50^ One method proposed for controlled and directed olfactory scent delivery has been to use ultrasound.^51^ This system however produces turbulent scent flow, with scent intensity decreasing with distance, a challenge also associated with many current air-based SDDs.^45^

Close-to-face SDDs are alternatives to air-based systems, with examples including:

- Multi-fragrance olfactory display – a small, light device consisting of cartridges that can release up to eight fragrances using controlled and precise scent delivery,^52^
- Direct-injection wearable olfactory display – a wearable device constructed of three units: an odour-presenting unit using an inkjet head device, a breath-detecting unit to detect the user's breathing pattern, and a control unit,^53^ and
- Wearable necklace (Essence) – a remote-controlled, olfactory computational necklace that can vary intensity and frequency of the released scent based on biometric or contextual data.^54^

A device-agnostic, software architecture has also been developed to help design scent experiences that can be connected to any device:

- Olfactory experience toolkit (OWidgets) – a toolkit comprising two units: a graphical user interface and device-independent software for olfactory experience design which instructs and manages the delivery of olfactory scents.^50^

The primary advantages of close-to-face SDDs are small dimensionality and portability. These smaller, sometimes miniaturised wearable devices, allow both researchers and participants to easily travel with the devices. It is also possible to facilitate the delivery of scents outside traditional laboratory settings, including domiciliary and working environments, allowing scent to be studied in various contexts, such as field and longitudinal studies.^45^

By combining digital healthcare and HCI technologies, the combination of objective measures with the more commonly used subjective responses, typically derived from hedonic and/or sliding scales and questionnaires, to determine the presence and severity of scent impairment for the clinical management of PD beyond research projects could be possible.^55,56^ Novel SDDs could also be used for other neurological conditions, such as Alzheimer's disease, where significant olfactory dysfunction and loss often go unnoticed.^57,58^

The aim of this pilot focus group study was to understand older people's perception of multisensory digital technologies specifically for scent training by defining the relationship between PD and scent dysfunction, defining HCI technologies and exploring scent-delivery devices and asking for initial thoughts for using a developed scent-delivery device as part of regular scent training to either attempt to recover some olfactory function and perhaps enable earlier diagnosis.

## Methodology

### Participant selection

Participants who were aged 60 years or above, diagnosed with PD and had the capacity to consent were included. Individuals were excluded from the pilot if they were unable to or did not provide consent. All participants were members of Parkinson's UK Research Support Network and received email invitations, prepared by the authors, directly from Parkinson's UK explaining the purpose of the pilot focus group interview. Written consent was obtained before the data collection period began.

### Ethical considerations

Approval was obtained from University College London's Research Ethics Committee (16717/002 – 15th June 2021). All participants were recruited voluntarily after providing consent, through email confirmation of participation and signing of consent forms. Confidentiality was maintained through secure and restricted access to the data to only the research team and immediate destruction of focus group recordings after transcription.

### Data collection and procedure

The pilot focus group was conducted in October 2021 over Zoom (Zoom Video Communications, Inc, San Jose, CA, USA) by a multidisciplinary research team, comprising three authors of the paper (N.D., M.Ob. and M.Or). The four members of the research team formulated possible discussion points surrounding the loss of sense of scent in PD and characterised these into three main themes:

1. Overview of sensory HCI technologies;
2. Real-world use of sensory HCI technologies to tackle problems encountered (i.e. concerns and/or an ability and willingness to use and/or help in development); and
3. Using sensory HCI technologies to meet specific requirements (i.e. scent training to sense, improve and/or treat loss of scent associated with PD).

The focus group was chaired by a facilitator (N.D.), who had previous experience in leading similarly sized group sessions. A semi-structured agenda, predetermined by the research team, was used to guide the focus group discussions; an introduction to HCI (M.Ob), an understanding of participants’ motivation to be involved in research (M.Or), exploration of the scientific relationship between PD and scent with relevant current advancements in HCI (M.Ob), understanding older persons opinions on the use of multisensory technologies for scent training (M.Ob.), since older people can be more vulnerable and often excluded in use of technology and gauging first impressions and thoughts on scent therapy and scent delivery for academic research in this sub-population (M.Or).

Participants were actively encouraged to present thoughts and opinions on the topics presented by the research team. From their responses, the facilitator, in consultation with the research team and participants, was able to prioritise specific points of interest and further development. The digital focus group lasted approximately two hours. The Zoom meeting was recorded and transcribed verbatim and members of the research team (M.Ob and M.Or) took additional notes during the focus group to capture essential information.

## Results and discussions

### Focus group participants

A total of 12 email responses from potential participants within Parkinson's UK Research Support Network were received. Two individuals were excluded as they were carers of patients with PD and a further two were excluded as they were below the minimum age of 60 years. The main reason provided for declining participation by the remaining five individuals was conflicting agendas; the COVID-19 pandemic had caused short-notice amendments to schedule appointments for these individuals who had initially expressed interest in and signed up to participate. The pilot focus group recruited three participants (see Table 1). None of the three participants experienced any issues with using and navigating the functionalities present within Zoom for the duration of the focus group.

**Table 1.** Pseudo-anonymised information of participants of the pilot focus group.

Participant	Age	Gender	Time since PD diagnosis	Time since loss of scent*
A	72	Female	5 years	>5 years
B	64	Female	2 years	10 years
C	67	Male	4 years	>4 years

*> defines a duration before PD diagnosis where there was a gradual loss of sense of scent.

### Theme 1: Overview of sensory HCI technologies

The participants were asked to provide their definitions of HCI at the beginning of the focus group (see Table 2). Importantly, the initial invitation distributed to the participants had the terms ‘Human–Computer Interaction’ and ‘multisensory technology’ embedded into the body of text but were not explained or defined for the purpose of facilitating the truest initial understanding of the terms when heard by lay audiences. Unsurprisingly, none of the three participants had heard of the term prior to attending the Zoom session, perhaps demonstrating not only the infancy of the discipline but also the lack of HCI awareness amongst the lay population.

**Table 2.** Participant definitions of human–computer interaction (HCI).

Participant	Definition of Human–Computer Interaction (HCI)
A	The use of robots to complete a task
B	A brain interaction which can increase memory by increasing storage
C	Seeing something with your own eye and processing it so that we come to know and recognise it in the future

**Figure 1.**
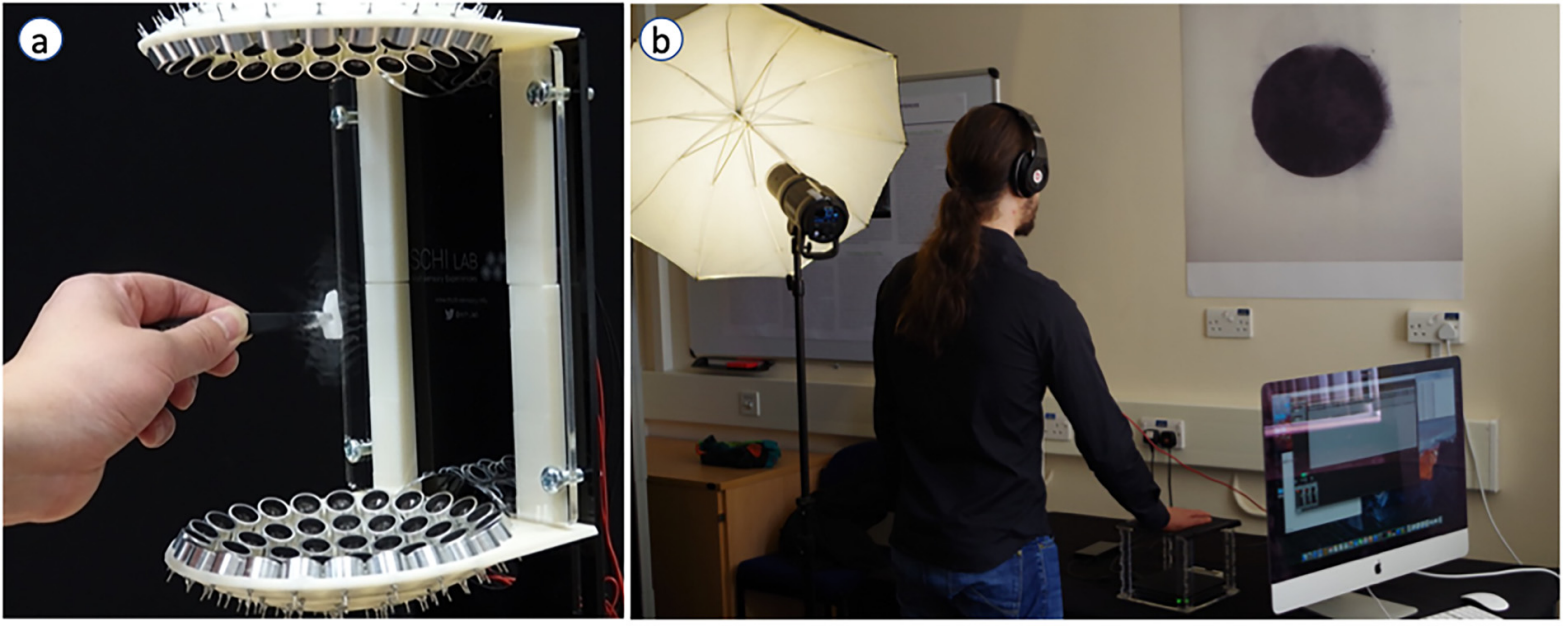
(a) The TastyFloats system that uses acoustic levitation to deliver food morsels to the users’ tongue (see further details in ref^59^) and, (b) Development of a multisensory art installation for Tate Britain (part of the Tate Sensorium project) where participants experience art not just through looking at it, but through feeling a painting via a haptic pattern projected on the user's right hand (see further details in ref^60^). Image Credit: M. Obrist/SCHI Lab.

During the initial discussions, the research team spoke of advancements in the HCI discipline over the past decades; the evolution of mobile phone and computer technology – examples that the focus group participants were likely familiar with. The participants were also shown examples of recent multisensory HCI technologies, developed and exhibited by the research team, to further demonstrate the power of the technologies within the discipline: TastyFloats, a novel system that uses acoustic levitation to deliver food ‘bites’ to the users’ tongue (see Figure 1(a)^59^), and the Tate Sensorium, a multisensory exhibition where visitors could interact with art via their own senses, especially touch, using mid-air haptic technology (see Figure 1(b)^60^).

### Theme 2: Real-world use of olfactory HCI technologies

Initial discussions during the pilot focus group were centred around the first instances where the three participants began to experience changes in their sense of scent. As shown in Table 1, decline in olfactory sense began gradually in the lead up towards their PD diagnosis, with loss of sense of scent occurring in the months and years to follow, as reported by the participants – 100% experienced olfactory dysfunction. Despite the low numbers recruited into the pilot focus group, the participants represent the higher end of percentage data regarding the prevalence of olfactory dysfunction in PD; from 45% to 49% in the first studies by Ansari and Johnson^61^ and Ward,^62^ up to 74% reported by Hawkes et al.,^63^ or 90–96% in the studies by Doty et al.^64^ and Haehner et al.^9^

Despite the onset of olfactory dysfunction, just one of the neurodegenerative symptoms present prior to the onset PD motor deficiencies,^65^ our participants found that healthcare professionals they visited talked down their concerns on loss of sense of scent (see Table 3). This represents the clear and obvious need to translate research studies into practice, such as the characterisation of potential biomarkers of preclinical PD (i.e. loss of olfactory function), which could help identify individuals who later develop disabling symptoms, making them ideal candidates for neuroprotective treatment strategies.^65^ Furthermore, loss of smell has gained momentum due to COVID-19, increasing awareness amongst the general public about the possible link between smell loss and their own health, be it a viral infection, or its association with neurogenetic deceases. Even having identified scent loss earlier, tests for olfactory function quantification must be normalised and standardised to accurately report statistical data^9^ and alleviate worries, as expressed by participant A, *‘at the moment, there's nothing that anyone has shown can actively make a difference, so do you want to know earlier, or is it just not more years worrying about it and thinking about the future*’.

**Table 3.** Participants on healthcare professional responses to the initial concerns expressed on loss of scent.

Participant	Comment
B	*I did feel the doctor dismissed it when I said I couldn't scent anything 10 years ago and didn't really explore or explain any other possibilities.*
C	*My consultant, a few [four]years ago, said it can’t be Parkinson's and most probably a virus, so that was the end of it.*

Of the three individuals, only participant B actively mentioned loss of scent to family and friends, whilst C only discussed the topic when provoked and A avoided the topic of conversation entirely when in the company of others. Whilst sharing health status is entirely an individual's preference, the later onset of olfactory dysfunction for participants A and C, in line with the time of PD diagnosis, may suggest an unawareness and under-recognition by healthcare professionals of scent impairment as an early maker of PD.^16^

Participants B and C were the only individuals taking medication at the time of the pilot focus group and both were adhering to their regimen. Neither reported any changes in olfactory function nor were they able to recall any instances of entering and/or leaving on-off phases associated with PD whilst on drug treatment. Interestingly, olfactory impairment has been reported to be linked to cholinergic transmission impairment,^8,66^ which may explain why no improvements were observed with levodopa, a dopa-decarboxylase inhibitor, in individuals with some scent dysfunction.^67^ Rasagiline, a monoamine oxidase type B inhibitor, was reported to allow significantly better odour sensing abilities in early-stage PD patients.^68^

The loss of speech, memory and movement in PD is all supported by training and rehabilitation programs. There, however, seems to be a clinical absence of olfactory training, despite studies from Haehner et al.^69^ and Knudsen et al.^70^ indicating that scent training may increase olfactory sensitivity in PD patients. None of the focus group participants had tried scent training, although participant A continues to ‘*try to scent flowers in the garden where possible*’. Olfactory training involves actively ‘sniffing’ the same scents twice daily over several months, where the scents chosen to represent one of four scent categories: flowery, fruity, spicy, and resinous.^71^ At the time of first diagnosis, participant C did join a single scent test for clinical research where ‘*petrol, cherry and pineapple scents*’ were offered for assessment, fitting the two of the four scent categories. Interestingly, the same participant *‘didn’t realise that I had lost my sense of scent before my diagnosis and that this was linked to Parkinson's. I couldn’t pinpoint a day or month when I lost my scent, but I know when I got my essential tremor*’. However, the three participants were open to scent training with the hope of regaining some olfactory function.

### Theme 3: Olfactory-based HCI technologies – individual expectations for future design of olfactory HCI technologies for PD

**Figure 2.**
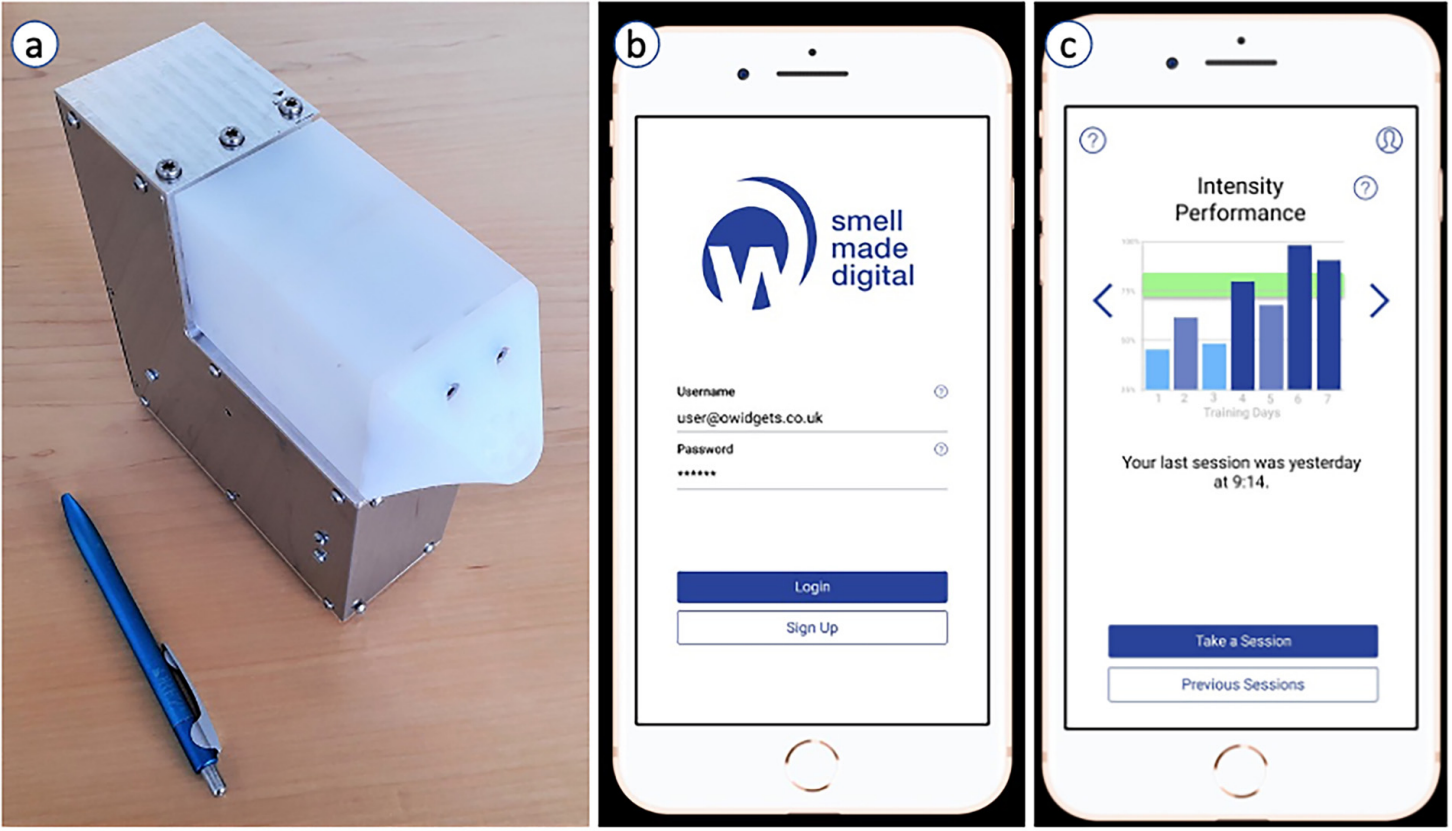
(a) The current prototype of a six-channel scent-delivery device (SDD) for digital smell training, with size comparison to a pen demonstrating portability, (b) simple log in/sign up landing page of the SmellHealth app coupled with the SDD, and, (c) Screenshot from a sample view in the mobile app which displays user performance and progression with each smell training session completed over time. Image Credit: E. Maggioni/OWidgets.

A redeveloped multichannel (six-channel) SDD (see Figure 2(a)), modelled on the OWidgets reference framework that had previously been designed and produced by the research team,^50^ was introduced to focus group participants. As individuals who were interest in adopting scent training into their routines to potentially regain some olfactory sensitivity, the value of the three participants’ thoughts and contributions on the SDD as a scent training aid towards the early diagnosis of PD proved invaluable.

Similar to the OWidgets toolkit,^50^ the new multichannel SDD enabled information (data instructions) to be transferred between an application (the graphical user interface) to an olfactometer device (using a *Mapper* and *Scheduler* to transmit instructions to the uniform device interface), followed by the release of a specific scent. The delivery of an in-person focus group would have enabled participants to observe live demonstrations on how to operate the new multichannel SDD and gain hands-on experience to provide specific feedback. However, the virtual session was designed to address this specific issue. Participant B was the first to comment, stating that ‘*the device looked quite straightforward actually*’. This sentiment was shared by C but was concerned by the device's large size when first presented.

The multichannel SDD was designed to mimic a printer, such that ‘scent cartridges’ can be installed into one of the many channels, with each ‘cartridge’ filled with a scent of the user's choosing. The choice of scent was welcomed by the focus group participants, with B preferring to ‘*have a range of scents to experience horrible scents as well as nice ones to trigger memories from the past that would otherwise be lost*’, whilst A wanted ‘*to choose the scents so as to leave the nasty scents behind*’. Initially, these ‘cartridges’ would be filled with scents of greater intensity (e.g. lemon, peppermint and eucalyptus) placed between those of lesser intensity (e.g. lavender and rose),^72^ since unlike light, sound and taste which are all spectral, scent is a chemical sense.^73^ The six-channel SDD was designed to be representative of the ‘odour prism’^74^ which describes six odour categories of flowery (rose), foul, fruity (lemon), aromatic (cloves), burnt and resinous (eucalyptus). The vast majority of olfactory training research uses four odours^75^ cloves, lemon, eucalyptus and rose and does not include the burnt and foul categories. However, with the current six-channel smell device prototype we were able to fully represent the original ‘odour prism’ and decide what additional custom odours could be included.

SDD design and technology continues to evolve, but as with any other user experience, the process can be subjective and can introduce variability within and between individual(s),^76,77^ making routine practice and adoption of scent training one of the greatest challenges – currently no part of our health culture and habit takes routine care of human olfaction.

To aid regular scent training, a two-step strategy has been incorporated into the SDD graphical user interface – generation of a user profile (a mobile app) and creation of a community. Similar to OWidgets, the user profile enables the individual to develop a personalised olfactory experience, detailing: individual preferences, trained scent-associations and scent-area,^50^ whilst accounting for basic demographic information. Data storage and visualisation allow users to view progress and enable the olfactory experience to be tailored (see Figure 2(c)), thus creating accurate experiences for specific individuals and/or user groups (e.g. Parkinson's disease).

Whilst initial scent training sessions with the multichannel SDD would be undertaken individually, with time, friends and/or family members could join the user's community and compare performances. Participant B was encouraged by this, commenting ‘*I think it would be a good idea if I did it with my daughter and we compare how we’re doing to one another. Having someone physically there to discuss things with each day and establish differences would help*’. With recent statistics suggesting that up to 25% of all PD cases are hereditary,^78^ PD patient's children participating in scent training may help to identify the potential onset of the disease earlier than anticipated. Although it must be mentioned that family members may be more inclined to scent train if benefits were already perceived and established by the primary user, rather than use it for themselves.

Two participants, specifically A and C, were asked whether a sense of community engagement using the mobile app would prompt them to share their scent loss experience with others. Interestingly, A, who previously avoided the topic of conversation entirely, even when prompted by their social circle, felt more inclined to share experiences within a community (see Table 4). This sense of community can be extremely powerful. The fitness and wellness industry has capitalised on user's wanting to compare and compete with like-minded individuals, often using gamification, to make strides towards their personal goals with Strava,^79^ Peloton,^80^ Apple Fitness+^81^ and Garmin Connect^82^ just some of the most popular apps available. Scent training, developed specifically for healthcare, will likely prioritise user comparison as opposed to competition and gamification to allow individuals to monitor their progression against family member or friends and/or peers with similar olfactory function, since monitoring potential PD progression is far more critical.

**Table 4.** Participant A on engaging with a community of individuals in a similar position.

Participant	Comment
A	*I think it's only really in that sort of setup that you feel really comfortable discussing it, otherwise it can easily sort of begin to sound like you're whinging or it feels negative. The self-support of discussing it with other people, I mean it's no different than when I first started a family you've got your concerns about a young baby, and so you get together with other people going through the same time and hope the help improves it a bit*

Overall, the responses from the three focus group participants for both scent training and the newly developed SDD were positive. All three expressed the sentiment that they ‘*would try to do anything that may bring some of those memories back*’, and even if some olfactory function was restored, they would try scent training ‘*as often as possible… up to 10 times a day if necessary*’. For PD patients, routine is fundamental to their day-to-day, especially given the frequency at which medicines are often administered. Based on the discussions from this focus group, PD patients could be inclined to incorporate scent training into daily routines, even if only minimal olfactory function was restored, subject to being able to interact with the newly developed SDD. Whilst there is no guarantee of this, the improvement in quality of life would far outweigh the time spent completing scent training.

The use of SDDs has been reserved for research purposes. However, all other body systems and organs can and are subject to regular examination and check-ups. Only recently, largely due to the COVID-19 pandemic, has scent training gained importance in domestic settings, with individuals using essential oils to test olfactory function.^83^ Scent training using SDDs should become a staple in routine healthcare practices. More importantly, regular use of SDD-assisted scent training from a younger age may facilitate earlier PD diagnosis, thus enabling earlier therapeutic and symptomatic disease management.

### Limitations

This pilot focus group study recruited and detailed only qualitative data from three participants. Whilst there was diversity in age, gender and experience of PD and olfactory dysfunctions, this minimal dataset collected is the greatest limitation to this study. Although the focus group was delivered virtually via Zoom and discussions between the participants and research team were rich, the inability to host the session in-person due to the COVID-19 pandemic limited how the participants viewed, were provided demonstration of and interacted with the multichannel device and associated mobile app. The research team has however planned to run an in-person follow-up session to provide more detailed information on the technologies.

## Conclusions

Digital healthcare technologies have been used in PD to monitor patients displaying motor signs and symptoms, with non-motor symptoms yet to receive the same attention. HCI, which uses computer technology to explore human activities and work, can be used in conjunction with digital technologies to design and develop tools to assess non-motor symptoms, including scent impairment, which often precedes motor symptoms in PD patients. Participants of an online pilot focus group were found to have experienced olfactory dysfunction in the years leading up to PD diagnoses; all now have complete loss of scent. Around the time of the participants’ diagnoses, scent impairment was not thought to be a critical non-motor symptom in the development of PD by the healthcare professionals visited.

Sensory HCI technologies, such as SDDs, have been developed in research environments to promote scent training. SDDs combine a multichannel device with a mobile app allowing users to assess and monitor individual scent function over time. Focus group participants expressed positive interest in regularly participating in scent training with the hope that some olfactory function could be restored, represented by visual improvements in the device-associated app and perhaps slight improvements in day-to-day scents. Participants would also encourage younger family members to join training sessions to aid early identification of scent impairment, as some PD cases are hereditary. Furthermore, those who did not currently discuss scent impairment with others were more likely to share experiences with a similar community. Whilst SDDs have been mostly limited to research settings, standardised devices for regular healthcare check-ups may facilitate an improvement in the olfactory senses of current PD patients and/or earlier PD diagnosis, allowing earlier therapeutic and symptomatic PD management by healthcare professionals. It may also be possible to extend the application of olfactory testing and SDDs to other neurodegenerative conditions, including Alzheimer's disease.
